# Effect of glucagon-like peptide-1 receptor agonists on cigarette smoking consumption in type 2 diabetes patients: study protocol of a randomized, parallel -controlled clinical trial

**DOI:** 10.3389/fcdhc.2026.1665837

**Published:** 2026-02-02

**Authors:** Da Chen, Ziyi Li, Chenxia Zhou, Ruoxuan Li, Xinnan Ji, Bo Feng, Jun Song

**Affiliations:** Department of Endocrinology, East Hospital, Tongji University School of Medicine, Shanghai, China

**Keywords:** cigarette smoking cessation, fMRI, functional magnetic resonance imaging, GLP-1RAs, glucagon-like peptide-1 receptor agonists, T2DM, type 2 diabetes mellitus, nicotine dependence

## Abstract

**Introduction:**

Glucagon-like peptide-1 receptor agonists (GLP-1RAs) are widely used for type 2 diabetes mellitus (T2DM) and may influence reward-related pathways, suggesting potential effects on nicotine dependence and smoking-related outcomes. Randomized evidence in patients with T2DM remains limited. This trial evaluates the effects of GLP-1RAs on nicotine dependence and smoking exposure and explores potential neural mechanisms using functional MRI (fMRI).

**Methods and analysis:**

This single-center, parallel-group randomized controlled trial will enroll 46 male adults with T2DM who are current smokers with Fagerström Test for Nicotine Dependence (FTND) score ≥4. Participants will be randomized (1:1) to receive a GLP-1RA or a dipeptidyl peptidase-4 inhibitor (DPP-4i) for 24 weeks as part of routine glucose-lowering therapy optimization. No structured smoking cessation counseling or smoking cessation pharmacotherapy will be provided by the research team. The primary endpoint is change in FTND score from baseline, assessed at weeks 1, 4, 8, 12, and 24. Secondary endpoints include changes in exhaled carbon monoxide (CO) and smoking cessation rate at weeks 12 and 24, and changes in metabolic parameters. Exploratory endpoints include changes in resting-state fMRI measures from baseline to week 24 and their associations with smoking- and metabolic-related outcomes.

**Trial status:**

Recruitment started in June 2025 and is ongoing.

**Clinical Trial Registration:**

ClinicalTrials.gov, identifier (NCT06924697).

## Introduction

Diabetes has emerged as an increasingly critical global health issue. In 2021, approximately 537 million adults worldwide were diagnosed with diabetes, and this number is projected to rise to 783 million by 2045 (1). Cigarette smoking is an important risk factor for the development of type 2 diabetes mellitus (T2DM) and contributes to poor glycaemic control as well as the progression of chronic diabetic complications. Smoking is independently associated with elevated hemoglobin A1c (HbA1c) levels and significantly increases all-cause mortality and cardiovascular risk among patients with diabetes (2).

Beyond its metabolic consequences, cigarette smoking is causally associated with chronic obstructive pulmonary disease, interstitial lung disease (3), impaired pulmonary function, and multiple malignancies (4, 5). Smoking also promotes systemic inflammation and immune dysfunction, further complicating the clinical management of patients with T2DM (6, 7). Although substantial progress has been made in tobacco control, smoking cessation success rates remain suboptimal, highlighting the need for novel strategies, particularly in metabolically vulnerable populations.

Glucagon-like peptide-1 receptor agonists (GLP-1RAs) are widely used in the treatment of T2DM due to their glucose-lowering efficacy and beneficial effects on body weight (1, 8). Emerging evidence suggests that GLP-1 signaling may influence addictive behaviors through central nervous system pathways (9). However, evidence regarding the effects of GLP-1RAs on smoking-related outcomes in patients with T2DM remains limited.

A recent large-scale real-world observational study using target trial emulation reported an association between semaglutide use and a reduced risk of tobacco use disorder in patients with T2DM (10). While this study provides important epidemiological evidence, randomized clinical trials incorporating objective behavioral measures and neurobiological endpoints are still lacking.

Therefore, this randomized controlled trial aims to investigate the effects of GLP-RA on nicotine dependence and smoking-related outcomes in patients with T2DM, and to explore potential neural mechanisms using functional magnetic resonance imaging (fMRI).

## Objectives

### Primary objective

To evaluate the effect of GLP-RAs on nicotine dependence in patients with type 2 diabetes mellitus, as assessed by changes in the Fagerström Test for Nicotine Dependence (FTND) score.

### Secondary objectives

To assess the effects of GLP-1RAs on smoking exposure, measured by exhaled CO levels, and on smoking cessation rate, as well as on metabolic parameters.

### Exploratory objectives

To explore changes in brain functional activity and connectivity in addiction-related regions using fMRI, and to investigate potential associations between neural measures, nicotine dependence, and metabolic changes.

## Methods and analysis

### Study design and setting

This two-arm randomized controlled trial is designed to evaluate the efficacy and safety of GLP-1RAs in cigarette smoking cessation compared with control treatment. Eligible participants who provide written informed consent will be randomly assigned in a 1:1 ratio to receive either a GLP-1RA or a DPP-4 inhibitor (DPP-4i). The study is being conducted at the Department of Endocrinology, Shanghai East Hospital, starting in June 2025. Participants are not required to have an intention to quit smoking at the time of enrollment.

**Figure 1:**
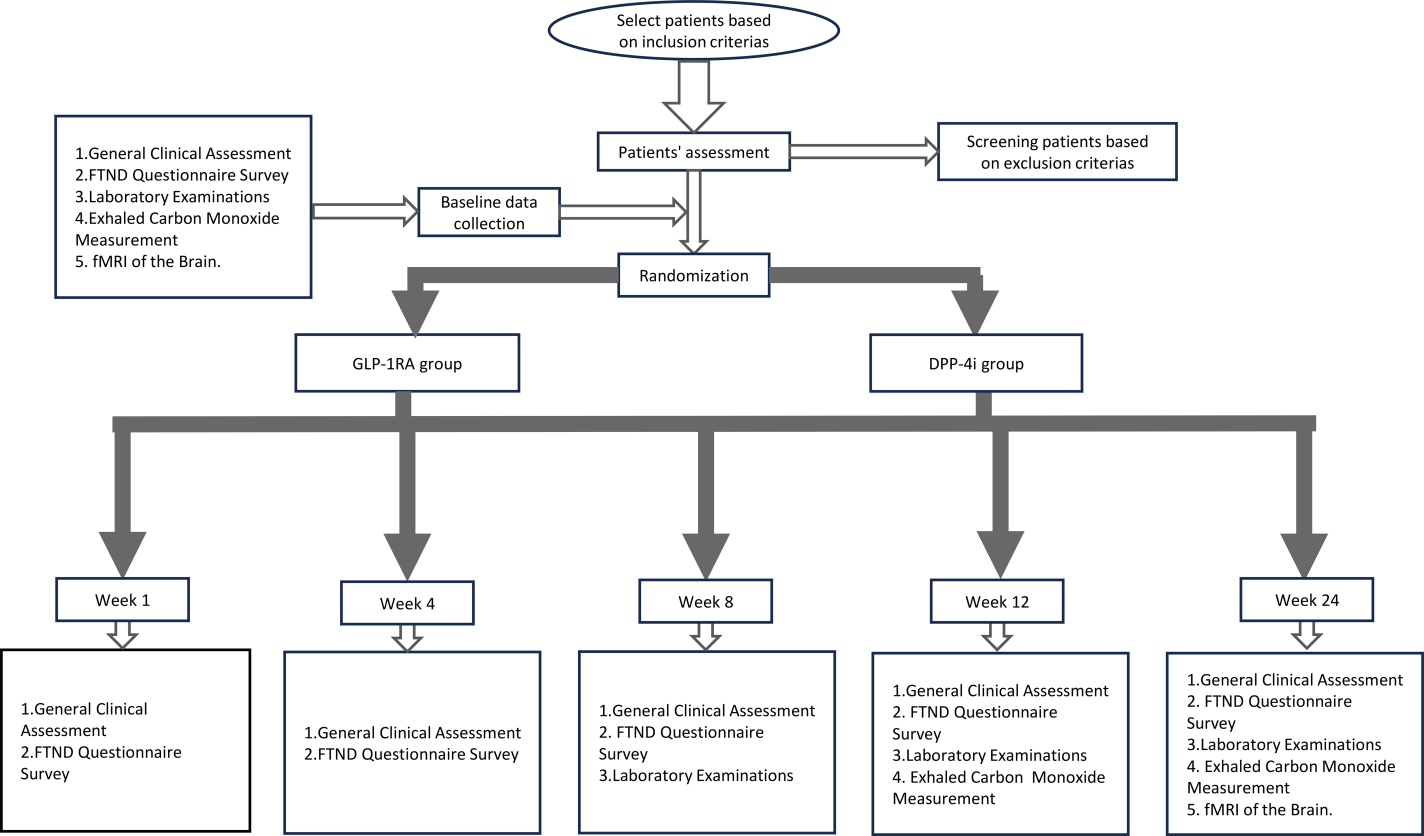
Flow chart of the study. GLP1-RA, Glucagon-Like Peptide-1 Receptor Agonist; DPP-4i, Dipeptidyl Peptidase-4 Inhibitor.

The research team will not provide any structured smoking cessation counseling or smoking cessation pharmacotherapy during the study period. Participants will undergo a structured intervention period, with follow-up assessments conducted at predefined time points to evaluate cigarette smoking cessation outcomes, nicotine dependence levels, and other relevant clinical parameters. All participants will be required to adhere to the follow-up plan as outlined in the study protocol. The study pathway is illustrated in **Figure 1**. The trial is registered at ClinicalTrials.gov (NCT06924697).

#### Recruitment

All participants will be recruited from the Department of Endocrinology at Shanghai East Hospital. Eligible participants are patients with T2DM who require adjustment or optimization of glucose-lowering therapy at baseline due to suboptimal glycaemic control or clinical indication for treatment modification. Participant selection will be based on the inclusion and exclusion criteria described below.

### Inclusion criteria

1. Male patients aged 18–75 years.
2. Diagnosis of T2DM based on the World Health Organization (WHO) criteria.
3. History of cigarette smoking for at least one year.
4. FTND score ≥4.
5. No contraindications to treatment with a GLP-1RA or a DPP-4i, and no prior use of these medications.
6. Ability to understand the study, voluntarily participate, and provide written informed consent.

### Exclusion criteria

1. Diagnosis of type 1 diabetes or other subtypes of diabetes.
2. Presence of diabetic ketoacidosis or severe diabetic complications.
3. Unstable or advanced cardiovascular, hepatic, renal, neurological, immunological, or hematological disease (acute heart failure, decompensated cirrhosis, end-stage renal disease). Presence of severe infection, malignancy, recent major surgery, or trauma.
4. History of severe recurrent hypoglycemia.
5. Severe gastrointestinal disorders (symptomatic gastroparesis) affecting drug absorption or tolerance.
6. Severe psychiatric disorders (schizophrenia, paranoid psychosis, bipolar disorder, intellectual disability).
7. Contraindications to MRI (metallic implants, pacemaker, severe claustrophobia).

### Withdrawal criteria

Patients may withdraw from the study at any time for the following reasons:

1. Withdrawal of informed consent.
2. Loss to follow-up.
3. Patient request for early termination of study participation.
4. Any other event deemed by the investigator to pose a significant risk to the participant or significantly impact study outcomes.

### Baseline assessment

After confirming eligibility and obtaining informed consent, patients will undergo a comprehensive baseline assessment. This includes recording demographic and anthropometric data (age, sex, and body mass index [BMI]) and a detailed medical history focused on diabetes and cigarette smoking status. Laboratory evaluations will include a complete blood count and routine blood chemistry (liver and kidney function tests, electrolytes, and coagulation profile), lipid panel, thyroid function tests, and measurements of HbA1c, insulin, and C-peptide levels. Urine samples will be collected for analysis of urine albumin-to-creatinine ratio (UACR). Exhaled CO levels will be measured, and the FTND questionnaire will be administered to assess baseline nicotine dependence. Additionally, each patient will undergo a baseline resting-state fMRI scan to assess functional connectivity in addiction-related brain regions.

### Study interventions

Participants in the experimental group will receive GLP-1RA treatment, while those in the control group will receive a DPP-4 inhibitor. The dosage for both groups will be determined according to standard therapeutic regimens for their diabetes treatment. In the GLP-1RA group, GLP-1RA therapy was prescribed in accordance with routine clinical practice and standard titration protocols. The GLP-1RA agents used in this study primarily included semaglutide and liraglutide. Semaglutide is initiated at 0.25 mg once weekly and titrated to a target maintenance dose of 0.5–1.0 mg once weekly, as tolerated. Liraglutide is initiated at 0.6 mg once daily and titrated to a target dose of 1.2–1.8 mg once daily. Dose adjustments are performed according to tolerability and routine clinical guidelines. The specific GLP-1RA agent and achieved maintenance dose will be recorded for each participant. To enhance participant retention, research staff will contact patients by phone prior to scheduled follow-up visits. The follow-up schedule for this trial is outlined in **Table 1**.

**Table 1:** Schedule of interventions and assessments.

Study procedures week	0	1	4	8	12	24
Study visit	V0	V1	V2	V3	V4	V5
Eligibility assessment	X					
Inclusion/randomization	X					
Cigarette smoking status/CO measurement	X				X	X
smoking cessation rate					X	X
Medical history	X					
BMI	X	X	X	X	X	X
FTND scores	X	X	X	X	X	X
Laboratory testing (blood and urine)	X				X	X
fMRI	X					X
Adverse events	X	X	X	X	X	X

BMI, Body Mass Index; FTND scores, Fagerström Test for Nicotine Dependence scores; fMRI, Functional Magnetic Resonance Imaging.

### General procedures

At baseline (week 0), informed consent will be obtained from all participants. After medical history collection and eligibility assessment, patients will be randomized to either the GLP-1RA group or the DPP-4i group. BMI will be recorded at weeks 0, 1, 4, 8, 12, and 24. Any adverse events occurring during the study period will be documented at each visit.

### Laboratory assessments

Blood samples will be collected at baseline, week 12, and week 24 for laboratory analysis. These tests will include complete blood count, liver and kidney function tests, electrolytes, coagulation profile, HbA1c, lipid panel, thyroid function, insulin, and C-peptide levels. Urine samples will be collected at baseline, week 12, and week 24 for UACR test.

### Questionnaire assessment

To evaluate cigarette smoking behavior and nicotine dependence, the FTND will be administered at baseline (week 0) and at weeks 1, 4, 8, 12, and 24. FTND scores will be recorded at each time point. **Table 2** presents the FTND questionnaire.

**Table 2:** Fagerstrom Test of Nicotine Dependence (FTND) scores.

Questions	Answers	Points
1.How soon after you wake up do you smoke your first cigarette?	Within 5 minutes	3
	6–30 minutes	2
	31–60 minutes	1
	After 60 minutes	0
2.Do you find it difficult to refrain from cigarette smoking in places where it is forbidden e.g.in church, at the library, in cinema, etc.?	yes	1
	no	0
3.Which cigarette would you hate most to give up?	The first one in the morning	1
	All others	0
4.How many cigarettes/day do you smoke?	10 or less	0
	11-20	1
	21-30	2
	31 or more	3
5.Do you smoke more frequently during the first hours after waking than during the rest of the day?	yes	1
	no	0
6.Do you smoke if you are so ill that you are in bed most of the day?	yes	1
	no	0

### Exhaled carbon monoxide measurements

Exhaled CO levels will be measured at baseline (week 0), week 12, and week 24.

### fMRI assessment

At baseline and 24 weeks, participants will undergo resting-state functional MRI scans to investigate treatment effects. MRI data will be acquired at Shanghai East Hospital using a 3.0 Tesla MRI scanner with a standard head coil. Resting-state functional images will be obtained using a gradient-echo echo-planar imaging (EPI) sequence with the following parameters: repetition time (TR) ≈ 2000 ms, echo time (TE) ≈ 30 ms, flip angle ≈ 90°, field of view ≈ 220 × 220 mm², matrix size ≈ 64 × 64, slice thickness ≈ 3.5 mm, and no inter-slice gap. High-resolution T1-weighted structural images will be acquired for anatomical reference. Resting-state functional connectivity (rsFC) analysis will be conducted to assess correlations in blood-oxygen-level-dependent (BOLD) signal fluctuations between different brain regions in the absence of explicit tasks (11–13). Changes in functional connectivity and brain structure will be analyzed in addiction-related regions, including the praecuneus, lingual gyrus, and paracentral lobule.

#### Primary outcome

The primary outcome is the change in the FTND score at weeks 1, 4, 8, 12, and 24.

#### Secondary outcomes

Secondary outcomes include changes in exhaled CO levels and smoking cessation rate at weeks 12 and 24. Laboratory assessments at baseline, week 12, and week 24 will include HbA1c, complete blood count, liver and kidney function tests, electrolytes, coagulation profile, lipid panel, thyroid function, insulin, and C-peptide levels, as well as UACR. Changes in body weight and BMI will be recorded at weeks1, 4, 12, and 24. Additionally, fMRI analyses will compare brain activity in addiction-related regions (praecuneus, lingual gyrus, paracentral lobule) between baseline and 24 weeks to explore the neural mechanisms underlying cigarette smoking cessation.

### Sample size calculation

The primary hypothesis is that at the 6-month follow-up, the GLP-1RA group will have a mean FTND score reduction of approximately 2 points compared with the control group. Based on previous randomized studies evaluating the effects of GLP-1 receptor agonists on smoking-related outcomes and nicotine dependence, the standard deviation of FTND scores was estimated to be approximately 2 points, and a 2-point reduction was considered clinically meaningful (14, 15). To detect this difference with 80% power at a 0.05 significance level, and accounting for an anticipated 10% dropout rate, 23 participants per group (46 total) are required. The anticipated dropout rate was set at 10%, considering that participants are receiving standard diabetes treatments within routine clinical care and that regular follow-up contacts are implemented to enhance retention. Even with a modestly higher dropout rate, the study would retain adequate power to detect clinically meaningful differences in FTND scores.

### Blinding and randomization

After obtaining informed consent and completing baseline assessments, participants will be randomly assigned in a 1:1 ratio to the GLP-1RA group or the control group. The randomization sequence will be generated by an independent statistician and sealed in sequentially numbered, opaque envelopes managed by an independent administrator. A study nurse or investigator not involved in outcome assessments will open the envelope to reveal each participant’s group allocation. Outcome assessors and data analysts will remain blinded until the database is locked for final analysis.

### Data management

All personnel involved in data collection, entry, the data manager, statistician, and outcome assessor, will receive training on data management procedures. Upon completion of the treatment phase, all participant information will be fully documented in the original case report forms (CRFs). Data will be collected and managed using an electronic data capture (EDC) system designed for clinical research. The system will support role-based access control, data validation rules, audit trails, and version control to ensure data quality and integrity. Source data verification and regular data monitoring will be conducted according to predefined procedures. Any data modifications will be tracked with timestamps and user identification.

All research-related paper documents will be securely preserved, while electronic files will be stored on a password-protected computer to ensure data security. Both paper and electronic records will be retained for at least five years after the study’s publication. If reviewers or readers have inquiries regarding the published data, they may contact the corresponding author to request access to the original dataset. Confidential patient information, such as names and phone numbers, will be strictly protected.

### Quality control

All participants and researchers should first receive training and complete the study, including follow-up steps. All the data will be strictly stored in the electronic database.

Throughout the research process, we will try our best to prevent the loss of participants. To ensure the punctuality and effectiveness of follow-up visits, we will communicate with patients by Social Networking Service or telephone during the study to improve patient compliance. Researchers will check the authenticity, accuracy, and completeness of the data obtained. After the validation is complete, the data will be locked for final data analysis. This series of data should be kept for no less than five years.

### Statistical analysis

An independent statistician will perform data analysis using SPSS v.27 on an intention-to-treat basis, with a two-sided significance level of 0.05. Baseline characteristics will be compared using t-tests or Mann-Whitney U tests for continuous variables and chi-square or Fisher’s exact tests for categorical variables. Primary endpoints (FTND scores, exhaled CO, and cigarette smoking cessation rates) will be analyzed using generalized estimating equations (GEE) or repeated measures ANOVA, adjusting for baseline values. Secondary endpoints (e.g. HbA1c, BMI, cigarette smoking urge scores, and fMRI-derived measures) will be analyzed using t-tests, Mann-Whitney U tests, or Spearman correlation analysis. Subgroup analyses will evaluate the effects of adjunctive cigarette smoking cessation medications while controlling for potential confounders (age, BMI, diabetes duration, etc.). Exploratory analyses will further examine the associations between changes in metabolic parameters and smoking-related outcomes to assess potential mediation effects. Missing data will be handled primarily using the GEE model, with sensitivity analyses via multiple imputation if needed. Adverse events will be summarized descriptively and compared using chi-square tests; reasons for withdrawal will be assessed qualitatively. fMRI data will be explored using Regional Homogeneity (ReHo) and rsFC analyses to assess local and whole-brain changes. ReHo will be computed using Kendall’s coefficient of concordance (KCC) for each voxel and compared using voxel-wise tests with multiple comparisons corrected. For rsFC, time series will be extracted from predefined seed regions, correlated with other voxels, transformed to z-scores, and compared between groups using a general linear model (GLM) with cluster-level correction (p<0.05). Regression analyses will control for potential confounders (e.g. cigarette smoking level, age, socioeconomic status).

### Adverse events

Safety assessments will encompass the reporting of adverse events and serious adverse events, alongside clinical laboratory evaluations, physical examinations, and follow-up assessments.

### Modification of the protocol

Any modifications to the study protocol that may influence study conduct, patient benefit, or safety—such as changes in study objectives, design, participant population, sample size, procedures, or major administrative aspects—will require a formal amendment. Such amendments will be reviewed and approved by the ethics committee before implementation and will be reported to the relevant health authorities in accordance with local regulations.

## Discussion

This study protocol describes a randomized controlled trial designed to investigate the effects of GLP-1RAs on nicotine dependence and smoking-related outcomes in patients with T2DM. By integrating behavioral assessments, metabolic measures, and neuroimaging endpoints, this trial aims to provide insight into the potential mechanisms through which GLP-1RAs may modulate addictive behaviors.

Glucagon-like peptide-1 (GLP-1) is a gut-derived hormone that has received increasing attention in recent years. GLP-1 is secreted by intestinal L cells in response to food intake and promotes satiety, reduces food consumption, and delays gastric emptying through actions on the hypothalamus (16). GLP-1RAs have been widely used for the treatment of T2DM (17). Their glucose-lowering mechanisms include glucose-dependent stimulation of insulin secretion, suppression of glucagon secretion, enhanced glucose uptake in skeletal muscle and adipose tissue, inhibition of hepatic gluconeogenesis, delayed gastric emptying, and appetite reduction (18).

The theoretical rationale of the present study is grounded in emerging evidence that GLP-1 signaling extends beyond metabolic regulation and may influence reward processing and addiction-related neural circuits (19, 20). Endogenous GLP-1 is produced in the nucleus tractus solitarius and projects to mesolimbic regions involved in reward and motivation (21). Preclinical and clinical studies suggest that activation of GLP-1 receptors may attenuate reward-related responses, providing a biologically plausible mechanism through which GLP-1RAs could affect nicotine dependence (22).

Importantly, this trial enrolls patients with T2DM receiving routine glucose-lowering therapy, thereby reflecting real-world clinical practice. Both GLP-1RAs and DPP-4is are guideline-recommended therapeutic options, supporting the ethical appropriateness of the study design (23). Participants are not required to have an intention to quit smoking, and no structured smoking cessation interventions are provided during the study. This design allows the evaluation of smoking-related changes associated with pharmacological treatment rather than behavioral modification.

Notably, weight loss and improved glycemic control may partially mediate or confound smoking-related outcomes (24). To address this issue, sensitivity and exploratory analyses will consider changes in BMI and HbA1c. Furthermore, concomitant therapies, such as sodium–glucose cotransporter 2 inhibitors, may indirectly influence smoking-related outcomes; these factors will be recorded and taken into account when interpreting the results.

Neuroimaging research indicates that chronic nicotine use can induce cognitive, structural, and functional alterations in the brain. Prior studies have identified the insula as a key region implicated in smoking behavior and nicotine dependence (25, 26). Cortical thickness in insular subregions has been reported to be negatively associated with nicotine dependence (27, 28), and nicotine dependence severity has been positively correlated with activation of the anterior and posterior insula in response to smoking-related cues (29). More recent work has shown negative associations between nicotine dependence and resting-state functional connectivity between insular subregions and the superior parietal lobule (30). Accordingly, the insula has been proposed as a potential neural target for interventions for nicotine dependence (25, 26). In addition, the precuneus has been suggested as a neuroimaging biomarker of nicotine addiction, with regional homogeneity (ReHo) metrics showing potential utility in distinguishing nicotine-dependent individuals (31, 32).

However, most previous studies were conducted in general smoking populations without metabolic comorbidities and relied primarily on behavioral outcomes (14, 15). It remains unclear whether GLP-1RAs exert similar or distinct effects in patients with T2DM, a subgroup with greater metabolic burden and potentially increased difficulty in smoking cessation. Moreover, prior research has rarely incorporated neuroimaging measures in a systematic manner to probe underlying brain mechanisms. Therefore, the present study employs rsFC and ReHo analyses to explore treatment-related changes in neural activity. Given the exploratory nature of neuroimaging analyses, findings will be interpreted cautiously and will primarily serve to generate hypotheses and inform future studies.

In summary, by focusing on patients with T2DM, extending follow-up duration, and integrating functional MRI assessments, this trial aims to address important gaps regarding the potential effects of GLP-1RAs on smoking-related behaviors and their neurobiological mechanisms. The results are expected to provide a basis for future larger-scale studies that include both sexes, incorporate longer follow-up, and adopt more definitive clinical endpoints.

### Strengths and limitations

·This is a single-center, randomized, parallel-group controlled trial evaluating the effects of GLP-1RAs on nicotine dependence and smoking-related outcomes in patients with T2DM.

·The 24-week follow-up is longer than that of most prior pharmacological studies assessing smoking-related outcomes, allowing evaluation of sustained changes in nicotine dependence and smoking exposure.

·The protocol integrates objective measures of smoking exposure and exploratory neuroimaging endpoints to investigate potential neurobiological mechanisms.

The absence of a placebo group may limit causal inference regarding the effects of GLP-1RAs on nicotine dependence and smoking-related outcomes.

·Smoking status and nicotine dependence are partially assessed using self-reported questionnaires, which may introduce reporting bias and affect measurement accuracy.

·More than one GLP-1RA agent is permitted within the intervention arm; therefore, the study is designed to evaluate class effects rather than molecule-specific differences.

·Only male participants are enrolled to reduce sex-related biological heterogeneity, which may limit the generalizability of the findings to female populations.
